# Can we improve cognitive function among adults with osteoarthritis by increasing moderate-to-vigorous physical activity and reducing sedentary behaviour? Secondary analysis of the MONITOR-OA study

**DOI:** 10.1186/s12891-018-2369-z

**Published:** 2018-12-21

**Authors:** Ryan S. Falck, John R. Best, Linda C. Li, Patrick C. Y. Chan, Lynne M. Feehan, Teresa Liu-Ambrose

**Affiliations:** 1 Faculty of Medicine, Aging, Mobility and Cognitive Neuroscience Laboratory, Djavad Mowafaghian Centre for Brain Health, Department of Physical Therapy, University of British Columbia, 212-2177 Wesbrook Mall, Vancouver, BC V6T 1Z3 Canada; 2 Faculty of Medicine, Arthritis Research Canada, University of British Columbia, Vancouver, Canada

**Keywords:** Physical activity, Sedentary behaviour, Cognitive function, Osteoarthritis

## Abstract

**Background:**

Preliminary evidence suggests osteoarthritis is a risk factor for cognitive decline. One potential reason is 87% of adults with osteoarthritis are inactive, and low moderate-to-vigorous physical activity and high sedentary behaviour are each risk factors for cognitive decline. Thus, we investigated whether a community-based intervention to increase moderate-to-vigorous physical activity and reduce sedentary behaviour could improve cognitive function among adults with osteoarthritis.

**Methods:**

This was a secondary analysis of a six month, proof-of-concept randomized controlled trial of a community-based, technology-enabled counselling program to increase moderate-to-vigorous physical activity and reduce sedentary behaviour among adults with knee osteoarthritis. The *Immediate Intervention* (*n* = 30) received a Fitbit® Flex™ and four bi-weekly activity counselling sessions; the *Delayed Intervention* (*n* = 31) received the same intervention two months later. We assessed episodic memory and working memory using the National Institutes of Health Toolbox Cognition Battery. Between-group differences (Immediate Intervention vs. Delayed Intervention) in cognitive performance were evaluated following the primary intervention (i.e., Baseline – 2 Months) using intention-to-treat.

**Results:**

The intervention did not significantly improve cognitive function; however, we estimated small average improvements in episodic memory for the Immediate Intervention vs. Delayed Intervention (estimated mean difference: 1.27; 95% CI [− 9.27, 11.81]; *d* = 0.10).

**Conclusion:**

This small study did not show that a short activity promotion intervention improved cognitive health among adults with osteoarthritis. However, the effects of increased moderate-to-vigorous physical activity and reduced sedentary behaviour are likely to be small and thus we recommend subsequent studies use larger sample sizes and measure changes in cognitive function over longer intervals.

**Trial registration number:**

ClinicalTrials.gov Protocol Registration System: NCT02315664; registered 12 December, 2014; https://clinicaltrials.gov/ct2/show/NCT02315664?cond=NCT02315664&rank=1

**Electronic supplementary material:**

The online version of this article (10.1186/s12891-018-2369-z) contains supplementary material, which is available to authorized users.

## Background

One new diagnosis of osteoarthritis (OA) occurs every 60 s, such that 9.6% of all men and 18.0% of all women over age 60 have symptomatic OA [1, 2]. Of those living with OA, 80% will have limitations in movement and 25% cannot perform their major daily activities of life [2]. The pain of OA is associated with 1) reduced physical function and mobility [3]; and 2) increased frailty and falls risk [4]. While total knee replacement is effective for end-stage OA [5], it does not uniformly restore joint function, and 20% of patients continue to experience pain [6].

Preliminary evidence also suggests OA is associated with an increased risk of cognitive decline and dementia [7]. Although the association between OA and dementia is still under investigation [8], animal models suggest that peripheral inflammation associated with OA may trigger neural inflammation and induce Alzheimer’s disease pathology—the most common form of dementia [9]. Given that the number of cases of OA and dementia are each increasing as the population of older adults continues to grow [10, 11], there is an urgent need for effective treatment strategies for OA symptoms since this may also help reduce dementia prevalence.

Two frontline strategies for improving OA symptoms are increasing moderate-to-vigorous physical activity and reducing sedentary behaviour [12–16]. Briefly, moderate-to-vigorous physical activity (MVPA) refers to any behaviour which incurs ≥3.0 metabolic equivalents (METs), while sedentary behaviour (SB) refers to any behaviour which incurs ≤1.5 METs and occurs while sitting or lying down. While OA is linked to declines in joint protective biomarkers such as lubricin and pituitary adenylate cyclase-activating polypeptide (PACAP), and increases in inflammatory markers and apoptotic signaling [17, 18], animal models of OA indicate that MVPA can 1) promote lubricin synthesis [19–21]; 2) down-regulate apoptotic signalling [19]; 3) down-regulate inflammatory markers of OA including interleukin-1 [21]; and 4) may stimulate the production of PACAP [22]. Increased MVPA can also improve strength and balance in adults with arthritis [23], reduce the risk of falls [24], and reduce OA symptoms such as pain, fatigue, and joint stiffness [25]. While less is known about how SB may impact the symptoms of OA, epidemiological evidence suggests that reduced SB is associated with better physical function in adults with OA—independent of MVPA time [12, 13].

There is also strong evidence that both high MVPA and low SB are neuroprotective [26, 27]. Animal models suggest that MVPA reduces pro-inflammatory markers and amyloid β protein levels in transgenic mice predisposed to Alzheimer’s disease [28], and human epidemiological data consistently indicates that MVPA is associated with better cognitive function and a lower risk of cognitive decline [27]. Greater amounts of SB may negatively impact the cellular mechanisms by which MVPA improves cognitive health [29], and may alter the connectivity of the brain such that cognitive function worsens with greater SB [30]. As such, current guidelines suggest that all adults should engage in ≥150 min of MVPA each week and limit discretionary SB as much as possible [26].

Given that 1) increasing MVPA and reducing SB promotes cellular mechanisms which reduce OA associated inflammatory and apoptotic responses [19–22, 28, 29]; and 2) OA associated inflammation and apoptotic signalling increases dementia risk [9], it is plausible that increasing MVPA and reducing SB is an effective frontline dementia prevention strategy for adults with OA. Unfortunately, the uptake of knowledge about the importance of MVPA and SB for controlling OA symptoms and reducing dementia risk has been slow. Among adults living with OA, 87% do not meet current recommendations of ≥150 min/week of MVPA [31], and people with OA spend 61% of all waking hours in SB [32]. Finding strategies to address this knowledge-to-action gap are thus greatly needed since increasing MVPA and reducing SB among adults with OA may provide benefits for both physical and cognitive health.

One promising strategy for increasing MVPA and/or reducing SB is consumer-available, wearable activity-monitoring technology. These devices present several distinct advantages as a MVPA promotion and SB reduction tool including: 1) adults typically perceive activity-monitors as useful [33]; 2) these devices incorporate multiple behavioural change strategies [34]; and 3) clinicians can readily use these devices to help promote behaviour change among their underactive patients [35]. Importantly, we recently determined that a wearable technology enabled counselling program for adults with knee OA increased MVPA by 25 min/day and improved OA symptoms [36]. Within this study, we included secondary measures to determine if increasing MVPA and/or reducing SB among adults with knee OA could also benefit cognitive function. Thus, the aim of the present paper is to determine whether this intervention to increase MVPA and reduce SB among adults with OA also improved cognitive function.

## Methods

### Study design

This study was a secondary analysis of *Monitor-OA*, a six month randomized controlled trial (RCT) examining the efficacy of a technology-enabled counselling intervention for increasing MVPA and reducing SB in people with knee OA [36]. The study occurred between November 1st 2015 and June 1st 2017. We used a randomized delayed-control design. In this study design, randomization determined the timing of when the intervention was provided (i.e., immediately vs. a 2-month delay).

### Participants

We recruited community-dwelling adults from Vancouver, British Columbia who had a physician confirmed diagnosis of knee OA, or passed two criteria for early OA: 1) aged 50+ years; and 2) experienced knee pain during the previous year lasting > 28 separate or consecutive days [37]. Participants were excluded if they 1) had been diagnosed with inflammatory arthritis, connective tissue diseases, fibromyalgia, or gout; 2) used anti-rheumatic drugs or gout medications; 3) had previously underwent knee arthroplasty, or were on the waitlist to receive total knee arthroplasty; 4) had suffered an acute knee injury in the past six months; 5) had a body mass index (BMI) of > 40 kg/m^2^; 6) had received a steroid injection or a hyaluronate injection in the last 6 months; 7) were using medications which impaired physical activity tolerance (e.g., beta blockers), or had an inappropriate level of risk for increasing their physical activity. Participants were also excluded if they did not have access to a computer in their home, or did not have a personal email address. Potential participants completed the Physical Activity Readiness Questionnaire (PAR-Q) [38]. If a potential risk was identified by the PAR-Q, physician confirmation was required to ensure the participant was able to be physically active without supervision of a health professional.

**Fig. 1:**
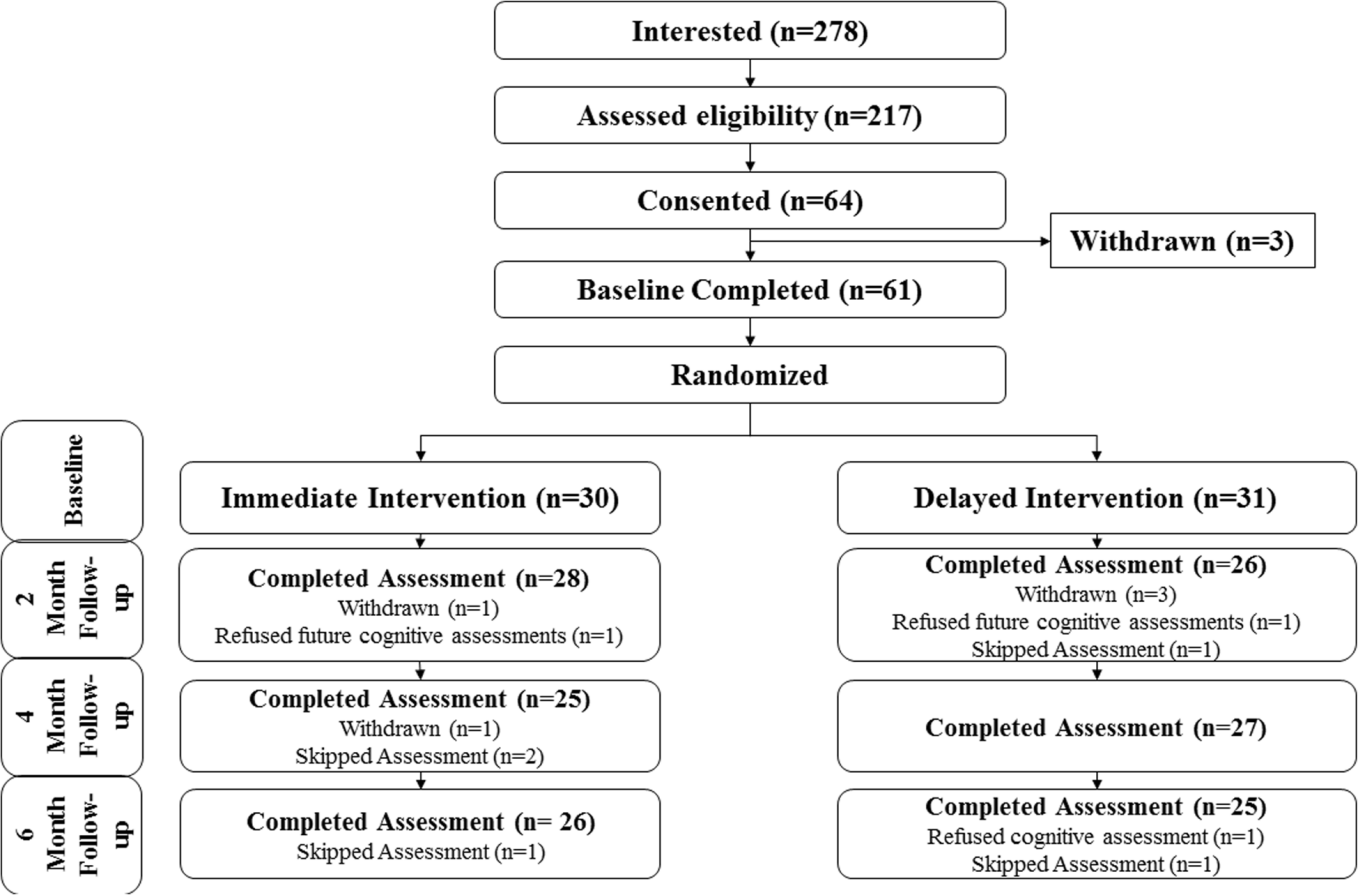
CONSORT Flow Chart

The CONSORT (Consolidated Standards of Reporting Trials) flowchart in Fig. 1 shows the number of participants in the treatment arms at each stage of the study [39]. The research protocol was approved by the University of British Columbia Behavioural Research Ethics Board (Application number: H14–01762), and was published in ClinicalTrials.gov (NCT02315664).

### Measures

Trained staff members administered all testing procedures. We assessed participants at baseline, 2 months, 4 months, and 6 months follow-up. In this paper we report data from baseline, 2 months, and 4 months.

#### Demographics

At baseline, we obtained general health history and demographics information by questionnaire. Height and weight were ascertained using a calibrated stadiometer and an electronic scale, respectively. This information was used to determine BMI. In addition, we assessed global cognitive function at baseline using the Mini-Mental State Exam and the Montreal Cognitive Assessment [40, 41].

#### MVPA and SB

We measured MVPA and SB using the SenseWear Mini (Body Media, Pittsburgh, PA, USA), a multi-sensor monitor worn on the upper arm over the triceps [42]. Briefly, the device integrates tri-axial accelerometer data, physiological sensor data and personal demographic information to provide valid and reliable estimates of MVPA and SB [42–44]. Participants wore the device on the non-dominant arm for 7 days at each assessment. For our analyses, we examined time spent in MVPA in periods of 10 or more minutes, and time spent in SB in periods of 20 or more minutes.

#### Cognitive function

We measured cognitive function using the National Institutes of Health Toolbox Cognition Battery (NIHTB-CB) [45]. Briefly, the NIHTB-CB provides a brief, convenient set of computerized and standardized measures of cognitive function. We examined two specific cognitive subdomains: 1) *episodic memory* using the picture sequence memory task [46]; and 2) *working memory* using the list-sorting task [47]. Empirical evidence suggests increasing MVPA or reducing SB can influence each of these domains of cognitive function [26, 48]. Briefly, the picture sequence memory task assesses episodic memory by having participants remember a sequence of actions embedded within a story. Participants re-arrange several pictures on the computer to match the sequence of events in the story. The list-sorting task assesses working memory by asking participants to repeat the names of orally—and visually—presented stimuli in order of size, from smallest largest. The number of items per set increases from one trial to the next and is discontinued once 2 trials of the same length are failed. Three trials of increasing length are completed. We recorded age-corrected scores for each measure.

### Randomization

After completing the baseline assessment, eligible participants were randomly assigned to the *Immediate Intervention* (I-INT) or the *Delayed Intervention* (i.e. control; D-INT) in a 1:1 allocation ratio. Randomization was performed using computer-generated random numbers in variable block sizes. The D-INT received the same intervention as the I-INT after a 2-month wait.

### Intervention

Details of the intervention have been described previously [36]. Briefly, the intervention consisted of participants attending a 1.5-h session, where they received: 1) a standardized group education session about the benefits of increasing MVPA and reducing SB; 2) a Fitbit® Flex™; and 3) individual activity counselling with a physiotherapist. The education session was delivered in groups of 2–3 participants. The individual activity counselling session followed the Brief Action Planning approach [49]. The physiotherapist guided participants to identify their MVPA goals (e.g., begin resistance training, start cycling, join a walking group, etc.), develop an action plan (i.e., where they plan to perform their MVPA goal, how often, and for how long), identify barriers and solutions, and then rate their confidence in executing the plan. For SB, the physiotherapists began by asking participants to estimate their sitting time in a normal day. Participants were then asked to identify ways to break up their sitting time into shorter bouts.

Following the education session, participants were provided with a Fitbit Flex™ which they were instructed to wear 24 h/day except during water-based activity (i.e., swimming or bathing) or when charging the device. The Fitbit data were wirelessly synchronized with Fitbit’s online Dashboard which could be viewed only by the participants and the study physiotherapist. During the intervention period, the physiotherapist reviewed the individual’s MVPA on the Dashboard and progressively modified the activity goals during 4 biweekly phone calls. During these phone calls, we also monitored participant adherence to SB goals using self-report. Specifically, participants were asked at each biweekly phone call whether they fully met, partially met, or did not meet their SB goal. These goals were then modified as needed.

### Statistical analyses

We conducted all statistical analyses using R version 3.3.1 in the *lsmeans 2.26–3, lmerTest 2.0–33,* and *mice 2.46.0* packages. Descriptive statistics were used to summarize participant demographics. In order to account for missing data at each follow-up time point, we performed multiple imputation in the *mice 2.46.0* package using predicted mean matching (5 imputations; 20 iterations each), and visually checked for convergence. All statistical models used pooled estimates from all 5 imputed data sets. Plots and graphs were created using *ggplot2 2.2.1.* Our statistical code can be found in Additional file 1.

#### Main analyses

We evaluated between-group differences (I-INT vs. D-INT) on the outcomes of interest following the primary intervention (i.e., Baseline – 2 Months) using the intention-to-treat principle, as per our primary outcomes paper [36]. Two separate analyses of covariance (ANCOVA) models were conducted, wherein cognitive performance at 2 months was the dependent variable and treatment group was the independent variable of interest; both models controlled for baseline cognitive performance. We estimated group mean changes in cognitive function and corresponding 95% confidence intervals pooled across the 5 imputed datasets, as well as estimated group mean difference (with 95% CI) and estimated Cohen’s *d* effect size.

#### Secondary analyses

We also examined whether changes in MVPA or SB during the intervention were associated with changes in cognitive function. We created change scores (i.e., I-INT = Baseline – 2 Months; D-INT = Baseline – 4 Months) for MVPA, SB, the list-sorting task, and the picture sequence memory task. We performed four separate multiple linear regression models wherein changes in cognitive performance (i.e., list-sorting memory or picture sequence memory) during the intervention were specified as the dependent variable, and changes in MVPA (or SB) was specified as the independent variable of interest. Each model included 1) baseline score for the cognitive performance variable of interest; 2) baseline MVPA (or SB); and 3) treatment group as covariates of no interest. We report unstandardized beta values and standard errors. Given our sample size, and a two-tailed α = 0.05, we had 80% power to detect a two-sided correlation with a medium effect size of |ρ| = 0.34 [50].

## Results

### Participant characteristics

From 2015 to 2016, 278 people indicated an interest to participate, and 64 met the eligibility criteria (Fig. 1). Of those, we recruited 61 participants (I-INT: *n* = 30; D-INT: *n* = 31). As described in Table 1, there were no group differences in age (I-INT: 61.73 [SD 9.40] years; D-INT: 62.61 [8.54]), sex (I-INT: 73.33% female; D-INT: 90.32%), BMI (I-INT: 29.16 [5.46] kg/m^2^; D-INT: 29.24 [4.82), or time spent in MVPA (I-INT: 83.44 [60.80] minutes/day; D-INT: 86.19 [86.19] minutes/day) and SB (I-INT: 681.96 [111.51] minutes/day; D-INT: 703.05 [161.17] minutes/day) at baseline. Participants in I-INT had a lower picture sequence memory score (I-INT: 102.04 [17.22]; D-INT: 112.53 [14.67]; *p* = 0.02), but there were no differences between groups for list-sorting score (I-INT: 102.05 [17.22]; D-INT: 102.42 [14.64]).

**Table 1:** Baseline Characteristics

Participant Characteristic	Immediate Intervention (*N* = 30)	Delayed Intervention (*N* = 31)	*p*
Age	61.73 (9.40)	62.61 (8.54)	0.70
%Female	73.33%	90.32%	0.16
Education			
*High school or less*	16.67%	19.35%	0.92
*Some university*	33.33%	29.03%	
*University degree or higher*	50.00%	51.61%	
Body Mass Index (kg/m)	29.16 (5.46)	29.24 (4.82)	0.95
Moderate-to-Vigorous Physical Activity (min/day)	83.44 (60.80)	86.19 (86.19)	0.89
Sedentary Behaviour (min/day)	681.96 (111.51)	703.05 (161.17)	0.55
Mini-Mental State Exam	28.03 (2.62)	28.62 (1.35)	0.28
Montreal Cognitive Assessment	27.27 (2.53)	26.24 (2.86)	0.15
List-Sorting Task	102.05 (13.03)	102.42 (14.64)	0.92
Picture Sequence Memory Task	102.04 (17.22)	112.53 (14.67)	0.02

### Changes in cognitive function

**Fig. 2:**
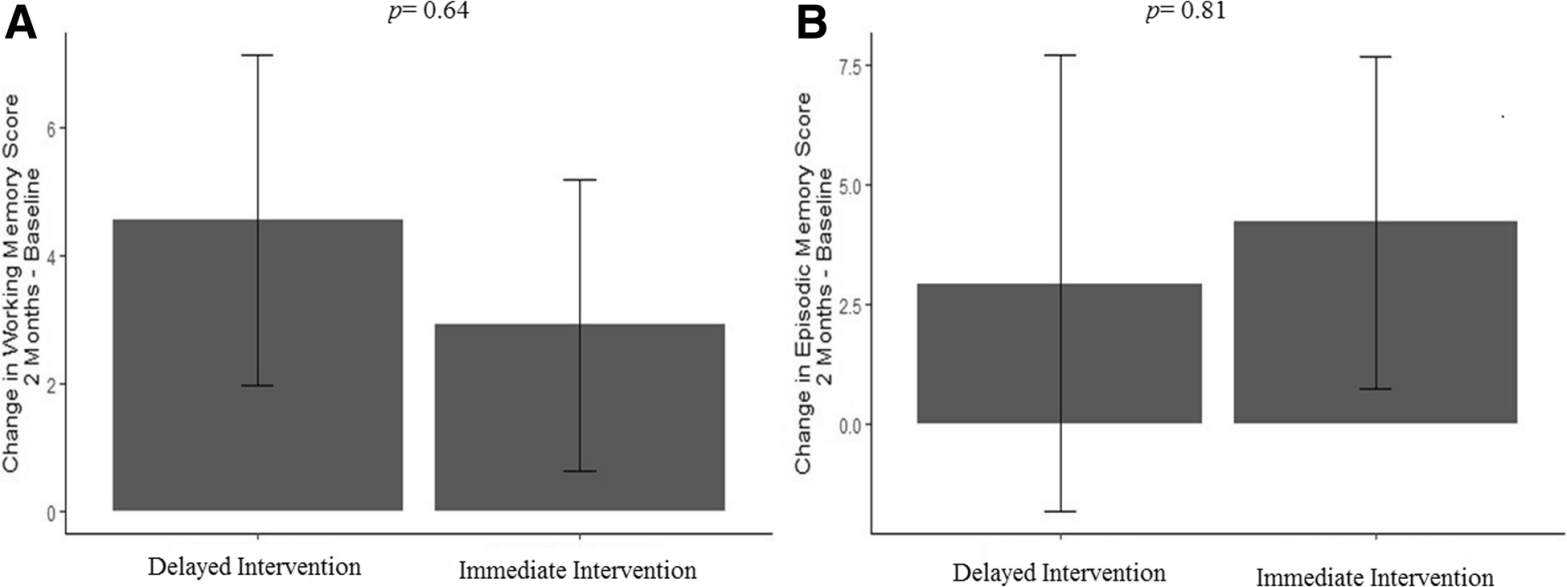
Changes in cognitive performance by treatment group (Baseline – 2 Months). **a** Change in NIH Toolbox List Sorting Task (i.e., Working Memory) score by treatment group adjusting for baseline cognitive score. **b** Change in NIH Toolbox Picture Sequence Memory Task (i.e., Episodic Memory) score by treatment group adjusting for baseline cognitive score

Group differences in cognitive performance—accounting for baseline cognitive performance—are illustrated in Fig. 2. Briefly, there were no statistically significant differences between groups following the intervention. As described in Table 2, we calculated a small improvement of the I-INT compared to D-INT for picture sequence memory (estimated mean difference: 1.27; 95% CI [− 9.27, 11.81]; *d* = 0.10), and a small improvement of the D-INT compared to the I-INT for list-sorting memory (estimated mean difference: -1.64; 95% CI [− 8.72, 5.44]; *d* = − 0.19).

**Table 2:** Changes in cognitive function (Baseline – 2 Months) by treatment group

Variable	Immediate Intervention Group Mean [95% CI]	Delayed Intervention Group Mean [95% CI]	Estimated Mean Group Difference [95% CI]	*d*
List Sorting Task	2.90 [−1.55, 7.35]	4.53 [−0.53, 9.59]	-1.64 [−8.72, 5.44]	−0.19
Picture Sequence Memory Task	4.21 [−2.55, 10.97]	2.95 [−6.36, 12.26]	1.27 [−9.27, 11.81]	0.10

*Note: All estimates adjusted for cognitive score at baseline*

### Correlation between MVPA and SB changes with changes in cognitive function

**Fig. 3:**
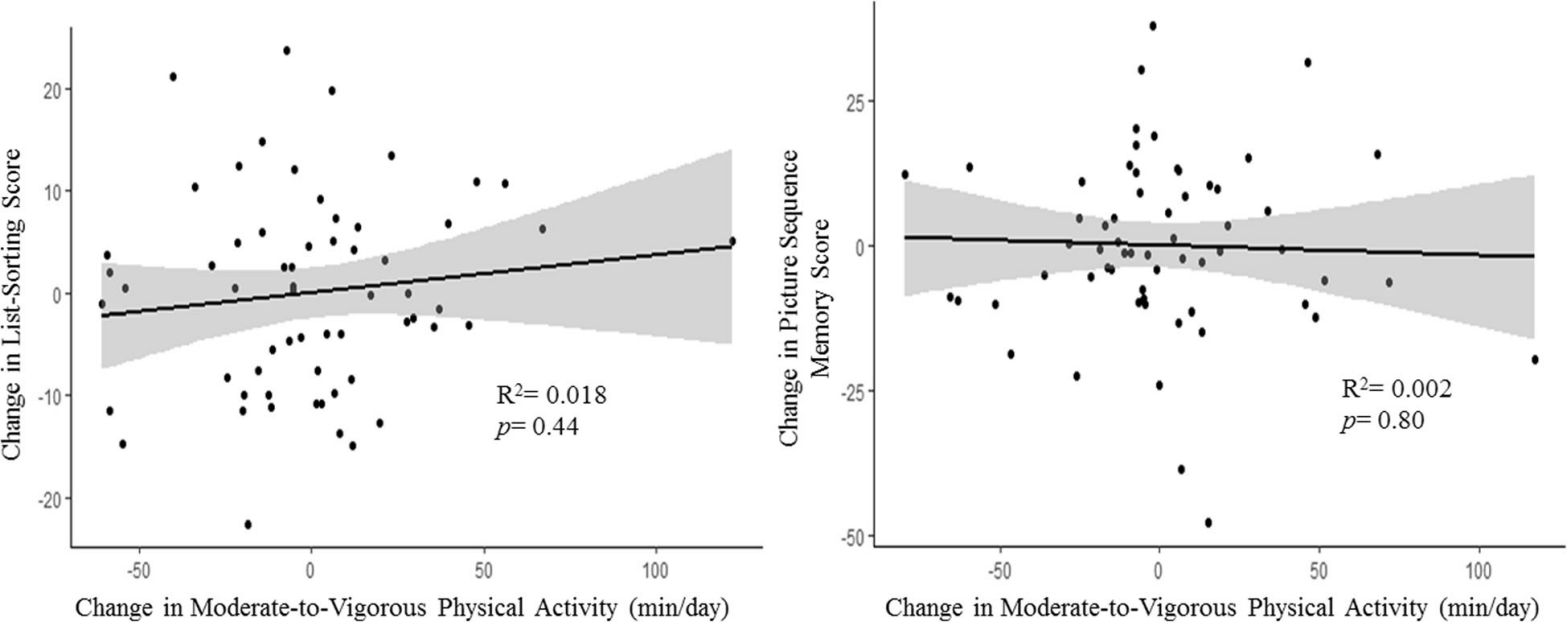
Relationship between intervention associated changes (i.e., *Immediate Intervention* = Baseline – 2 Months; *Delayed Intervention* = Baseline – 4 Months) in moderate-to-vigorous physical activity (minutes/day) and changes in cognitive function. Each model includes 1) baseline score for the cognitive performance variable of interest; 2) baseline moderate-to-vigorous physical activity; and 3) treatment group as covariates of no interest

The relationship between changes in MVPA and changes in cognitive function are illustrated in Fig. 3. There were no statistically significant relationships between changes in MVPA and changes in cognitive function. Increases in MVPA were correlated with changes in list-sorting memory in the expected direction (B = 0.04; 95% CI [− 0.07, 0.14]), however changes in picture sequence memory appeared to be negatively correlated with increases in MVPA (B = − 0.02; 95% CI [− 0.15, 0.12]).

**Fig. 4:**
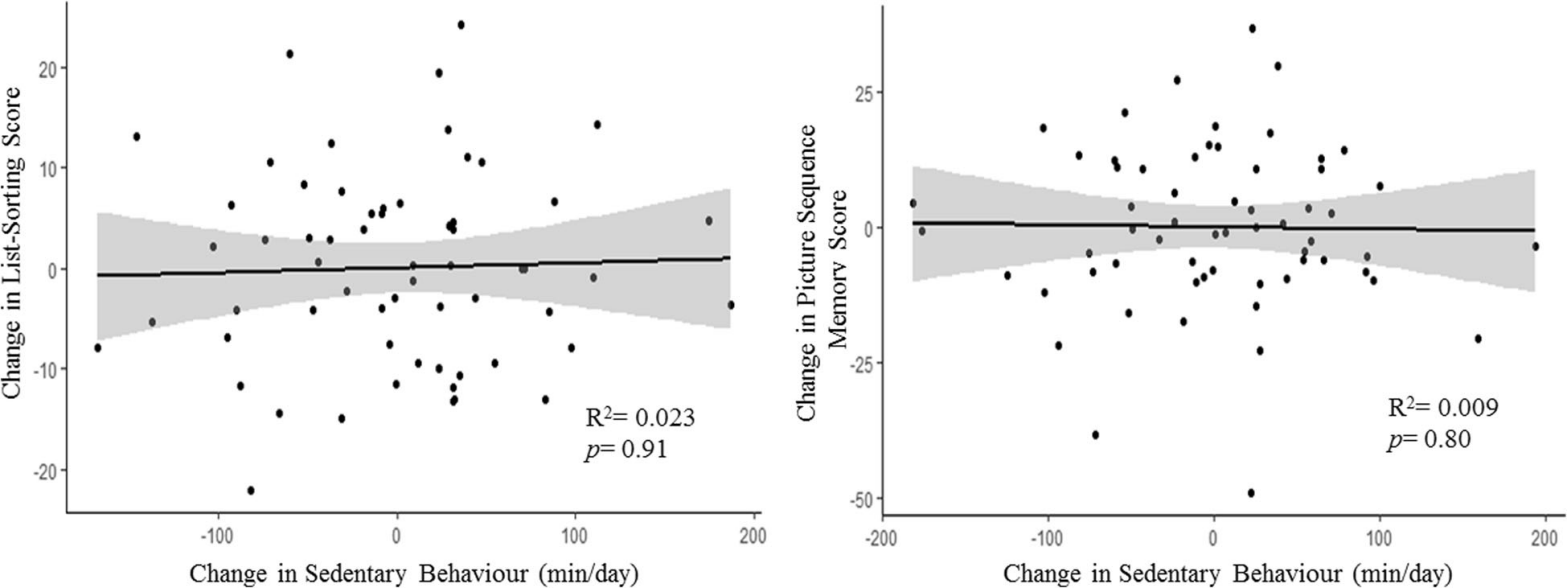
Relationship between intervention associated changes (i.e., *Immediate Intervention* = Baseline – 2 Months; *Delayed Intervention* = Baseline – 4 Months) in sedentary behaviour (minutes/day) and changes in cognitive function. Each model includes 1) baseline score for the cognitive performance variable of interest; 2) baseline sedentary behaviour; and 3) treatment group as covariates of no interest

The relationship between changes in SB and changes in cognitive function are illustrated in Fig. 4. There were no statistically significant relationships between changes in SB and changes in either picture sequence memory (B = − 0.01; 95% CI [− 0.09, 0.07]) or list-sorting memory (B = 0.00; 95% CI [− 0.09, 0.10]).

## Discussion

Although we previously determined this intervention can increase MVPA and improve quality of life among adults with knee OA [36], there does not appear to be sufficient evidence that our intervention can also improve cognitive function within this population. However, our results suggest that future research on the role of MVPA and SB for maintaining cognitive health among adults with OA is warranted. Given that most adults with OA are inactive [32], and thus more at risk to future cognitive decline [26, 27], we believe that such research would be valuable. We now discuss potential explanations for our findings.

While there is strong evidence that MVPA in the form of exercise training can improve cognitive function [51], the results of community-based MVPA interventions to promote cognitive health have been far less conclusive [52]. Importantly, the effects of MVPA on cognitive function seem to be largest for individuals who are underactive, while regular MVPA may be mainly neuroprotective for individuals who are meeting current guidelines [53]. Our participants were already highly active at baseline, and thus changes in MVPA may have had limited impact on cognitive function due to high basal levels. Moreover, the high activity level of our sample at baseline suggests that the results may not be generalizable to most adults with OA—who are sedentary. In order to first determine the efficacy of MVPA as a treatment for maintaining cognitive health for adults with OA, future trials should therefore recruit less active individuals since they 1) are more generalizable to the OA population; and 2) will more likely reap the most benefits from increasing their MVPA.

To date, most of the evidence examining how SB impacts cognitive health comes from epidemiological data [26, 29, 30]. Our study is the first to examine if an intervention to reduce SB can improve cognitive health among adults with OA. The results do not appear to suggest reductions in SB are associated with improvements in cognitive function; however, our intervention did not significantly reduce time spent in SB [36]. Preliminary evidence does suggest reductions in sedentary time may reverse the deleterious physiological effects of SB—such as impaired glucose and lipid metabolism [54]. Healthy glucose and lipid metabolism are strongly linked to better cognitive health [55]. Thus, while our results cannot determine whether reducing SB can improve cognitive function, there does appear to be a plausible mechanism by which reduced SB may benefit cognitive function.

This was a secondary analysis of a proof-of-concept RCT, and thus we think the logical next step is for an adequately powered RCT to determine the efficacy of this intervention to promote cognitive health among adults with OA. A recent meta-analysis suggests that MVPA in the form of exercise training has a modest effect size on cognitive function of *d* = 0.29 [51]. Based on this effect size, we post-hoc investigated the necessary sample size to perform an adequately powered RCT to improve cognitive function using G*Power 3.1 [50]. In order to detect an effect size of the magnitude suggested by Northey and colleagues [51], we would need at least 376 participants to achieve 80% power (assuming a two-sided α = 0.05). The study we report herein was thus under-powered, however we would expect the effect sizes for this intervention to increase through two simple strategies. First, future studies should exclude adults that are already physically active since the largest effects of MVPA on health occur for individuals who are inactive [56]. Second, increasing the length of time between assessment points would help reduce the potential for practice effects to occur on cognitive tests [57], and provide adequate time for eliciting changes in cognitive function which evidence suggests are larger after at least 6 months of increased MVPA [48].

### Clinical applications

Although we did not find that our intervention significantly improved cognitive function, there are several potential clinical applications to our study. First, we previously demonstrated that clinicians (i.e., physical therapists) can use consumer-available wearable activity-monitors such as a Fitbit to promote MVPA and reduce SB among their patients with OA [36]. Given the health care system has an untapped capacity for promoting changes in MVPA and SB, which to date has not been fully developed [58], a first step towards harnessing this potential to promote behaviour change is for clinicians to track their patients’ MVPA and SB using wearable activity-monitors. At minimum, clinicians should query about activity during their consultations with OA patients [59].

Secondly, both OA and SB may increase the risk of cognitive impairment and dementia [7–9, 26, 29, 30]. In contrast, engagement in MVPA reduces dementia risk and promotes overall cognitive and physical health [14–16, 27, 28]. Adults with OA should therefore be encouraged to adhere to the current public health recommendations of engaging in ≥150 min/week of MVPA and limiting their SB as much as possible [26]. Importantly, 87% of adults with OA are underactive [31] and adults with OA spend an average of 61% of the day in SB [32]. MVPA promotion and SB reduction may thus be particularly important for physical and cognitive health in adults with OA.

### Limitations and future research

This was a secondary outcomes analysis, and thus our findings are a preliminary investigation of whether increasing MVPA and reducing SB can improve cognitive function in adults with OA. While the SenseWear Mini provides valid and reliable estimates of energy expenditure for both younger and older adults [42, 60], which can be used to derive time spent in MVPA and time spent in SB, we cannot determine whether time estimated as SB was actually spent sitting or lying down. Hence, future studies to examine changes in SB should use measures of body posture such as the activPAL [61], which can accurately determine whether a person is sitting, standing, or walking.

We did not collect information on medication use, however our participants were community-dwelling adults who were healthy enough to start a physical activity program at study entry. We also did not exclude participants based on current activity level and hence the results may not be generalizable to most people with OA—who are often sedentary. Given the high activity level of our participants, the effects of increased MVPA and reduced SB on cognitive function may have been attenuated. We therefore suggest future studies should recruit underactive adults with OA, since these individuals are likely to benefit from increased MVPA and reduced SB.

There is growing evidence that the effects of MVPA (and potentially SB) are moderated by age and sex [62, 63], however due to our small sample size, it was not feasible for us to control for age and sex within our analyses. As detailed previously [36], our intervention did not reduce SB which is perhaps due to several shortcomings of the counselling program, which we have rectified. Specifically, the intervention now includes a new SB counselling strategy, and a Fitbit-compatible web app with enhances functionality for setting goals and rewarding behaviours that break up prolonged sitting [64]. This paradigm is currently being tested in a RCT (ClinicalTrial.gov identifier: NCT02554474) involving people with rheumatoid arthritis and systematic lupus erythematosus [65].

## Conclusion

While strong evidence indicates that increasing MVPA and reducing SB can positively impact OA symptoms, it is not yet clear whether increasing MVPA and reducing SB can also promote cognitive health among this population. However, increasing MVPA and reducing SB among adults with OA should be a public health priority since it can help maintain physical health and reduce the risk of cognitive impairment and dementia. Clinicians should therefore take the time to counsel their patients with OA to engage in ≥150 min/week of MVPA and limit their SB in order to promote physical and cognitive health.

## Additional file

Additional file 1: R Code. (DOCX 15 kb)

## Abbreviations

BMI: Body Mass Index
D-INT: Delayed Intervention
I-INT: Immediate Intervention
METS: Metabolic Equivalents
MVPA: Moderate-to-Vigorous Physical Activity
NIHTB-CB: National Institutes of Health Toolbox Cognition Battery
OA: Osteoarthritis
PACAP: Pituitary Adenylate Cyclase-Activating Peptide
PAR-Q: Physical Activity Readiness Questionnaire
RCT: Randomized Controlled Trial
SB: Sedentary Behaviour
