# Supplementary material for: Can we improve cognitive function among adults with osteoarthritis by increasing moderate-to-vigorous physical activity and reducing sedentary behaviour? Secondary analysis of the MONITOR-OA study
Source: BMC Musculoskelet Disord. 2018 Dec 21;19:447. doi: 10.1186/s12891-018-2369-z (PMC6303889; doi:10.1186/s12891-018-2369-z)
Supplement: Supplementary file 1 — R Code. (DOCX 15 kb) [file 12891_2018_2369_MOESM1_ESM.docx]

**Supplementary Material S1: R code**

1. **Data Management**

####LOAD PACKAGES####

if (!require("pacman")) install.packages("pacman")

pacman::p_load(readxl, plyr, psych, lme4,nlme, robustlmm, car, lsmeans,lmerTest)

library(emmeans)

####PREPARE DATA SET####

Monitor_Final_Long_Dec2017 <- read.csv("Monitor_Final_Long_Dec2017.csv")

Monitor_Final_Long_Dec2017$Tx<-NA

Monitor_Final_Long_Dec2017$Tx[Monitor_Final_Long_Dec2017$Treatment.Group==1]<-"Immediate"

Monitor_Final_Long_Dec2017$Tx[Monitor_Final_Long_Dec2017$Treatment.Group==2]<-"Delayed"

data1<-Monitor_Final_Long_Dec2017[c(1,313,6:9,15:16,18,94,170,246,20,96,172,248,25,101,177,253,28,104,180,256,

32,33,108,109,184,185,260,261,76,78,80,82,83,152,154,156,158,159,228,230,232,

234,235,304,306,308,310,311,36:38,40,43,46,41,44,47,112:114,116,119,122,117,120,123,

188:190,192,195,198,193,196,199,264:266,268,271,274,269,272,275)]

data1<-rename(data1,c("WM.AgeCorrectedScore.1"="WMAgeCorrectedScore.1",

"WM.AgeCorrectedScore.2"="WMAgeCorrectedScore.2",

"WM.AgeCorrectedScore.3"="WMAgeCorrectedScore.3",

"WM.AgeCorrectedScore.4"="WMAgeCorrectedScore.4",

"PSM.AgeCorrectedScore.1"="PSMAgeCorrectedScore.1",

"PSM.AgeCorrectedScore.2"="PSMAgeCorrectedScore.2",

"PSM.AgeCorrectedScore.3"="PSMAgeCorrectedScore.3",

"PSM.AgeCorrectedScore.4"="PSMAgeCorrectedScore.4",

ID_number<-substr(data1$ID,4,7)

ID_number<-as.vector(ID_number)

ID_number<-as.numeric(ID_number)

data1$ID_number<-ID_number

data2<-reshape(data1,idvar="ID_number",varying=c(9:88),direction="long")

data2$time<-data2$time-1

data2$timefactor<-as.factor(data2$time)

data2$Edu <- NA

data2$Edu[data2$Education=="Associates Degree"]<- "Some College"

data2$Edu[data2$Education=="Bachelors degree"]<- "University or Higher"

data2$Edu[data2$Education=="High School Graduate"]<- "High School or Less"

data2$Edu[data2$Education=="High Scool Graduate"]<- "High School or Less"

data2$Edu[data2$Education=="Masters degree"]<- "University or Higher"

data2$Edu[data2$Education=="None"]<- "High School or Less"

data2$Edu[data2$Education=="Postgraduate degree"]<- "University or Higher"

data2$Edu[data2$Education=="Primary School"]<- "High School or Less"

data2$Edu[data2$Education=="Some College"]<- "Some College"

data2$Tx<-relevel(as.factor(data2$Tx),ref="Immediate")

1. **Baseline descriptive statistics**

####BASELINE VALUES#####

Baseline <- subset(data2, time == 0)

table(Baseline$Tx, Baseline$Sex)

table(Baseline$Tx, Baseline$Race)

table(Baseline$Tx, Baseline$Edu)

describeBy(Baseline$Age, Baseline$Tx)

describeBy(Baseline$Age, Baseline$Tx)

describeBy(Baseline$MVPA_Min, Baseline$Tx)

describeBy(Baseline$SB_Min, Baseline$Tx)

describeBy(Baseline$PSMAgeCorrectedScore, Baseline$Tx)

describeBy(Baseline$WMAgeCorrectedScore, Baseline$Tx)

##Baseline t-tests##

t.test(Baseline$Age~Baseline$Tx)

t.test(Baseline$PSMAgeCorrectedScore~Baseline$Tx)

t.test(Baseline$WMAgeCorrectedScore~Baseline$Tx)

t.test(Monitor_Final_Long_Dec2017$BMI.1~Monitor_Final_Long_Dec2017$Treatment.Group)

t.test(Baseline$MVPA_Min~Baseline$Tx)

t.test(Baseline$MMSE~Baseline$Tx)

t.test(Baseline$MoCA~Baseline$Tx)

##Baseline Chi Square##

tbl <- table(Baseline$Sex, Baseline$Tx)

cbind(tbl,prop.table(tbl))

chisq.test(tbl)

tbl <- table(Baseline$Edu, Baseline$Tx)

cbind(tbl,prop.table(tbl))

chisq.test(tbl)

1. **Imputation and ANCOVA Models using Multiple Imputation**

##IMPUTATION 5 data sets, 20 iterations

tempdata <- mice(final.data, m=5, maxit=20)

summary(tempdata)

##IMPUTATION CHECKS##

#~~~~~~~~~~~~~~~~~~~~

tempdata$imp$SB_Min.3

tempdata$meth

plot(tempdata)

##SUMMARY OF MI MODELS###

##~~~~~~~~~~~~~~~~~~~~~~~##

#Imputation 1

tempdata1 <- complete(tempdata, 1)

tempdata2 <- complete(tempdata, 2)

tempdata3 <- complete(tempdata, 3)

tempdata4 <- complete(tempdata, 4)

tempdata5 <- complete(tempdata, 5)

##Working Memory ANCOVA Model using MI##

#~~~~~~~~~~~~~~~~~~~~~~~~~~~~~~~~~~~~~~#

WM.ANCOVA.MI.delay <- with(tempdata, lm((WMAgeCorrectedScore.2-WMAgeCorrectedScore.1)~scale(WMAgeCorrectedScore.1,center=TRUE,scale=FALSE) + Tx))

summary(pool(WM.ANCOVA.MI.delay))

WM.ANCOVA.MI.immediate <- with(tempdata, lm((WMAgeCorrectedScore.2-WMAgeCorrectedScore.1)~scale(WMAgeCorrectedScore.1,center=TRUE,scale=FALSE) + txr))

summary(pool(WM.ANCOVA.MI.immediate))

WM.ANCOVA.MI <- with(tempdata, lm(WMAgeCorrectedScore.2~WMAgeCorrectedScore.1 + Tx))

summary(pool(WM.ANCOVA.MI))

##Picture Sequence Memory ANCOVA model using MI#

##~~~~~~~~~~~~~~~~~~~~~~~~~~~~~~~~~~~~~~~~~~~~~##

PSM.ANCOVA.MI.delay <- with(tempdata, lm((PSMAgeCorrectedScore.2-PSMAgeCorrectedScore.1)~scale(PSMAgeCorrectedScore.1,center=TRUE,scale=FALSE) + Tx))

summary(pool(PSM.ANCOVA.MI.delay))

PSM.ANCOVA.MI.immediate <- with(tempdata, lm((PSMAgeCorrectedScore.2-PSMAgeCorrectedScore.1)~scale(PSMAgeCorrectedScore.1,center=TRUE,scale=FALSE) + txr))

summary(pool(PSM.ANCOVA.MI.immediate))

PSM.ANCOVA.MI <- with(tempdata, lm(PSMAgeCorrectedScore.2~PSMAgeCorrectedScore.1 + Tx))

summary(pool(PSM.ANCOVA.MI))
